# Highly stable graphene-oxide-based membranes with superior permeability

**DOI:** 10.1038/s41467-018-03919-0

**Published:** 2018-04-16

**Authors:** Khalid Hussain Thebo, Xitang Qian, Qing Zhang, Long Chen, Hui-Ming Cheng, Wencai Ren

**Affiliations:** 1 Shenyang National Laboratory for Materials Science, Institute of Metal Research, Chinese Academy of Sciences, 72 Wenhua Road, Shenyang, 110016 China; 2 University of Chinese Academy of Sciences, 19 A Yuquan Road, Beijing, 100049 China; 3 School of Materials Science and Engineering, University of Science and Technology of China, 72 Wenhua Road, Shenyang, 110016 China; 4 Tsinghua-Berkeley Shenzhen Institute (TBSI), Tsinghua University, 1001 Xueyuan Road, Shenzhen, 518055 China

## Abstract

Increasing fresh water demand for drinking and agriculture is one of the grand challenges of our age. Graphene oxide (GO) membranes have shown a great potential for desalination and water purification. However, it is challenging to further improve the water permeability without sacrificing the separation efficiency, and the GO membranes are easily delaminated in aqueous solutions within few hours. Here, we report a class of reduced GO membranes with enlarged interlayer distance fabricated by using theanine amino acid and tannic acid as reducing agent and cross-linker. Such membranes show water permeance over 10,000 L m^−2^ h^−1^ bar^−1^, which is 10–1000 times higher than those of previously reported GO-based membranes and commercial membranes, and good separation efficiency, e.g., rhodamine B and methylene blue rejection of ~100%. Moreover, they show no damage or delamination in water, acid, and basic solutions even after months.

## Abstract (web summary)

Graphene oxide membranes show great potential for water filtering, but improving their performance and stability remains difficult. Here, the authors use theanine amino acid and tannic acid to reduce and cross-link graphene oxide membranes with remarkably high permeability and stability in aqueous solution.

## Introduction

Nanofiltration (NF) membranes with nanometer-sized pores have attracted increasing interest because of a wide range of applications in desalination, water purification, pharmaceuticals, and petroleum chemistry^1–3^. Graphene oxide (GO)^4^ is a 2D network consisting of oxidized sp^3^ zones hybridized with polar oxygen functional groups and pristine graphitic sp^2^ zones^5,6^. In GO membranes, the oxidized zones act as spacer to provide relatively large interlayer distance to accommodate water molecules and the pristine graphitic zones facilitate rapid water permeability by nearly frictionless flow^7^. At the same time, the tunable subnanochannels between the neighboring GO nanosheets can act as molecular sieves, blocking all solutes with hydrated radii larger than the size of subnanochannels^8^. Therefore, GO membranes have been demonstrated to have a great potential as NF membranes for water purification, desalination, and molecular separation^9–20^. In addition, GO membranes are easy to fabricate, mechanically robust, and amenable to industrial-scale production with low cost^21,22^. Recent studies show that the water permeability of GO membranes can be improved by controlling subnanochannels and introducing more polar functional groups^23^.

However, it is challenging to further improve the water permeability without sacrificing the separation efficiency. Moreover, the GO membranes suffer from low stability in aqueous medium^9,24^. When the GO membranes are immersed in aqueous medium, the negatively charged GO sheets will separate from each other due to the electrostatic repulsion, leading to severe damage, and delamination of GO membranes within a few hours. Although it has been reported that the stability of GO membranes in water can be improved by using cross-linking reagents containing multivalent metal cations^25,26^ such as Al^3+^, the membranes are easily damaged in acidic and basic solutions^11^. Recent studies show that the chemical reduction by using hydroiodic acid^27^ or hydrazine^15^ can improve the stability of GO membranes in solution. However, the water permeability decreases drastically due to the narrower interlayer spacing between the reduced GO (rGO) sheets^14^.

Here, we report a class of rGO membranes with enlarged interlayer distance fabricated by using GO sheets and tannic acid (TA, Supplementary Fig. 1a) and theanine amino acid (TH, Supplementary Fig. 1b) as reducing agent and cross-linker. Such membranes show ultrahigh water permeability, good separation efficiency, and excellent stability. For instance, the rGO–TH membranes show water permeance over 10,000 L m^−2^ h^−1^ bar^−1^, which is 10–1000 times higher than those of the previously reported graphene-based membranes^14–20^ and commercial NF membranes^28^, and good separation efficiency, e.g., rhodamine B (RB) and methylene blue (MLB) rejection of ~100%. Moreover, they show no damage or delamination in water, acid, and basic solutions even after months. In addition, we demonstrated a green method to fabricate such membranes by simply using GO sheets and green tea (GT) extractives that contain TA and TH.

## Results

### Synthesis and characterization of rGO–TA and rGO–TH membranes

**Fig. 1:**
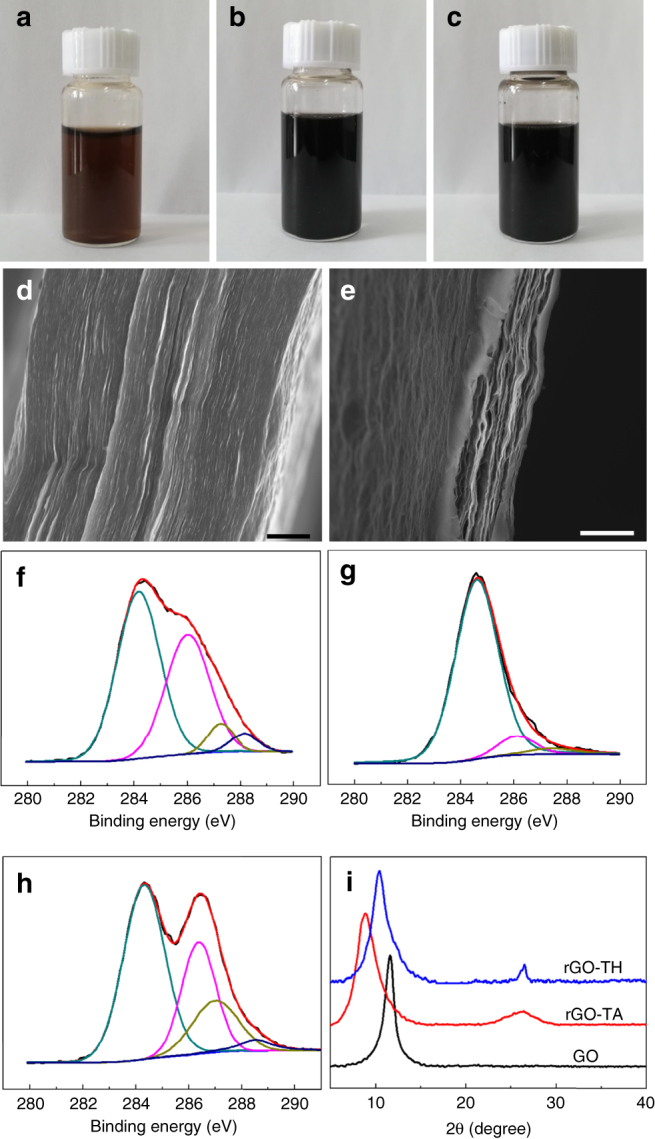
Structure characterization of GO-based membranes. **a**–**c** Photos of GO dispersion (**a**), rGO–TA dispersion (**b**), and rGO–TH dispersion (**c**). **d, e** Typical cross-sectional SEM images of GO (**d**) and rGO–TH membranes (**e**). **f**–**h** C 1 s XPS spectra of GO (**f**), rGO–TH (**g**), and rGO–TA (**h**) membranes. **i** XRD patterns of GO, rGO–TA, and rGO–TH membranes. Scale bars: (**d**) 1 μm; (**e**) 5 μm

GO sheets were synthesized by exfoliation of graphite oxide made by the modified Hummers method^29^ using sonication. They are monolayers with a thickness of ~1 nm and lateral size mostly in the range 0.5–1.5 μm (Supplementary Fig. 2). Before membrane fabrication, the poly(ether sulfone) (PES) substrate was modified with polydopamine to enhance its adhesion with the GO-based membrane^16^. To fabricate rGO–TH (or rGO–TA) membranes, certain amount of GO and TH (or TA) with a weight ratio of 1:1 were first fully dispersed/dissolved in DI water by sonication and then stirred overnight at 70 °C for reaction. Different from the brown color of GO dispersion (Fig. 1a), the rGO–TH and rGO–TA dispersion show black color (Fig. 1b, c), indicating the reduction of GO sheets. The dispersion was then filtrated through the polydopamine modified PES membrane in a vacuum filtration cell. For comparison, GO membranes were also fabricated on modified PES substrates through vacuum filtration of GO dispersion. The thickness of membrane was controlled by the content of GO and TH (or TA) in the dispersion. Different from the tightly stacked layered structure of GO membranes (Fig. 1d), the rGO–TH and rGO–TA membranes show porous layered structure (Fig. 1e).

TA molecule (C_76_H_52_O_46_) is very rich with hydroxyl groups (Supplementary Fig. 1a), and TH molecule (C_7_H_14_N_2_O_3_) contains both amino and oxygen groups (Supplementary Fig. 1b). We used X-ray photoelectron spectroscopy (XPS) to identify the chemical composition of the membranes. As shown in Fig. 1f, the GO membrane shows similar XPS spectra with those of GO reported^4–6^, which contains four components that correspond to the carbon atoms in hydroxyl, epoxy, carbonyl, and carboxyl groups. It is important to note that although TA and TH are rich with oxygen functional groups, which, respectively, have an O/C atomic ratio of 0.61 and 0.43, the O/C atomic ratio decreases from 0.44 for pristine GO membrane to 0.40 for rGO–TA membrane and 0.22 for rGO–TH membrane. These results give strong evidence that the GO has been reduced, which is consistent with the color change mentioned above. In particular, the rGO–TH membrane shows typical C1s XPS characteristic of rGO (Fig. 1g), with greatly reduced peak intensity related to the oxygen functional groups. Because of the high O/C atomic ratio of TA molecules (0.61), the rGO–TA membranes show similar C1s XPS characteristic with GO (Fig. 1h).

We then used X-ray diffraction (XRD) to characterize the structure of the GO, rGO–TA and rGO–TH membranes. As shown in Fig. 1i, GO membranes show a single diffraction peak at 2θ of 11.6°, corresponding to an interlayer distance of 0.76 nm, attributed to the oxygen containing functional groups and the trapped water molecules between the stacked GO sheets. Interestingly, rGO–TA and rGO–TH membranes show two XRD peaks, which are different from the reported rGO membranes^6,30^. The small XRD peaks at around 26.4° are similar to those observed in graphite, which might be related to the narrowed interlayer distances of neighboring rGO sheets that are attracted each other by π–π interaction caused by the removal of oxygen functional groups during reduction. More importantly, rGO–TA and rGO–TH membranes also show strong diffraction peaks at 2θ of ~8.9° and 10.4°, respectively, corresponding to the interlayer distance of 0.99 nm and 0.85 nm. The large interlayer distance implies that the TA or TH molecules are inserted between the neighboring rGO sheets as spacer in most the regions of the rGO–TA and rGO–TH membranes.

Fourier transform infrared (FTIR) spectroscopy was used to identify the cross-linking between rGO sheets and TA/TH molecules (Supplementary Fig. 3). Compared to GO membranes, rGO–TA membranes show two strong new peaks at 1025 and 1718 cm^−1^, which correspond to the stretching vibration of the –C–O–C moiety and –C=O moiety in ester groups (–O–C=O), respectively^31^. These two peaks are also observed in TA molecules but show much weaker intensity. The increased peak intensities indicate that the residual carboxyl groups of rGO have covalently bonded with the hydroxyl groups of TA molecules, leading to more ester groups. Similarly, rGO–TH membranes show much stronger ester C–O stretch peak at 1389 cm^−1^. Moreover, the peak at 2357 cm^−1^, which is assigned to the –OH stretching of –COOH in TH molecules^32^, is absent in rGO–TH membranes. These confirm that the –COOH groups of TH molecules have been covalently bonded with the residual oxygen functional groups of rGO nanosheets. Therefore, there exists strong covalent interaction between rGO sheets and TA or TH molecules in the rGO–TA and rGO–TH membranes. In addition, there should also exist hydrogen bonding interactions between rGO sheets and TA/TH molecules and π–π interactions between rGO sheets and TA molecules.

The above characterizations suggest that the rGO–TH and rGO–TA membranes are a class of layered structure materials, which are composed of rGO membrane domains with both normal and enlarged interlayer distance. Due to the removal of oxygen functional groups, such membranes have greatly increased graphitic regions compared to GO membranes. First, the greatly increased pristine graphitic regions and enlarged interlayer distance in the expanded domains can facilitate rapid water permeance by nearly frictionless flow^7^. Second, the strong π–π attraction in the normal domains as well as the strong covalent bonding between rGO sheets and TA/TH molecules in the expanded domains are helpful for suppressing the movements of the rGO sheets. Third, the stacked rGO sheets together with the inserted TA/TH molecules can act as molecular sieves, blocking the solutes with hydrated radii larger than the size of nanochannels^8^. Therefore, it is expected that the rGO–TA and rGO–TH membranes have ultrahigh water permeance, good separation efficiency, and high stability in various aqueous solutions.

### Stability of rGO–TA and rGO–TH membranes in aqueous solutions

**Fig. 2:**
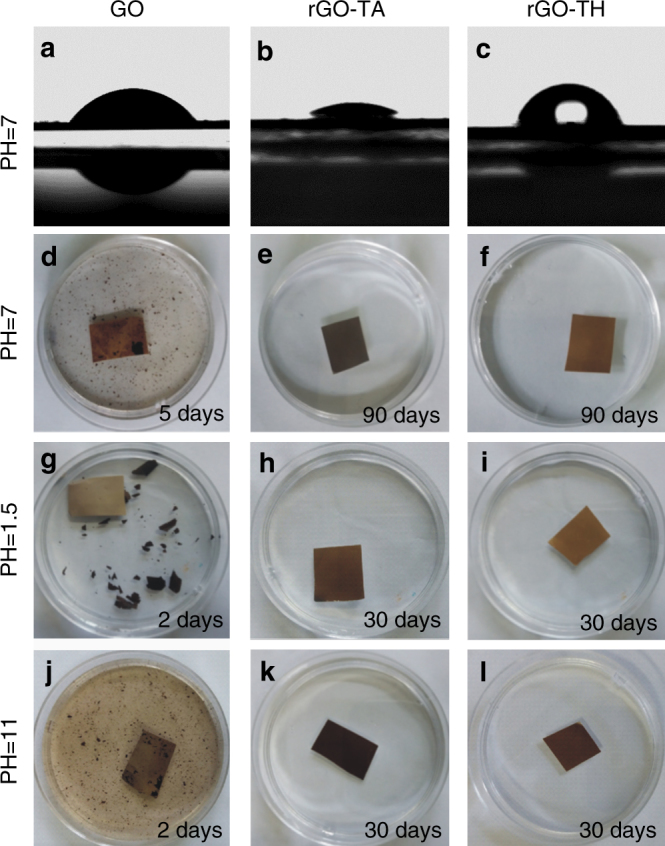
Hydrophilicity and stability of GO-based membranes. **a**–**c** Photos of a water droplet on GO (**a**), rGO–TA (**b**), and rGO–TH (**c**) membrane surface. **d**–**l** Stability in water (**d**–**f**), acidic solution (**g**–**i**), and basic solution (**j**–**l**). **d, g, j** GO membrane. **e, h, k** rGO–TA membrane. **f, i, l** rGO–TH membrane. The time staying in the aqueous solution is indicated in each photo

We first measured the hydrophilicity of GO, rGO–TA, and rGO–TH membranes at 23 °C and 33% of humidity. As shown in Fig. 2a–c, although GO sheets are reduced by TA and TH molecules, the rGO–TA membrane (contact angle, ~26°) shows much better hydrophilicity than GO membrane (contact angle, ~54°), and the rGO–TH membrane is also hydrophilic with a contact angle of ~73°. As we know, the GO membranes easily swell in water because of the uptake of water, which significantly influences the separation performance and cycling stability. We then evaluated the water uptake of GO, rGO–TA (50 wt%) and rGO–TH (50 wt%) membranes according to equilibrium weight swelling ratio (ESR). As shown in Supplementary Table 1, the swelling of rGO–TA (ESR, ~1.3) and rGO–TH (ESR, ~1.8) membranes are much less than that of GO membrane (ESR, ~2.2), indicating that the movements of rGO sheets are suppressed in the rGO–TA and rGO–TH membranes.

As mentioned above, the GO sheets tend to separate from each other in aqueous solutions due to the presence of oxygen functional groups, leading to damage and delamination of GO membranes. Therefore, long-term stability is essentially important for the solution-phase applications of GO membranes. We studied the stability of GO, rGO–TH, and rGO–TA membranes in different aqueous solutions. Similar to those reported previously, the GO membranes are readily disintegrated in water after 5 days (Fig. 2d). In contrast, the rGO–TA and rGO–TH membranes are very stable and remain their original structure even after 90 days (Fig. 2e, f). More importantly, the rGO–TA and rGO–TH membranes are also very stable in acidic (pH = 1.5, Fig. 2h, i) and basic (pH = 11, Fig. 2k, l) solutions, which is sharply different from the GO (Fig. 2g, j) and modified GO membranes reported so far^11,25,26^. As shown in Fig. 2g, j, the GO membranes are delaminated without shaking or stirring after 2 days in acidic and basic solutions, while no visible damage or delamination is observed for the rGO–TA and rGO–TH membranes even after 30 days (Fig. 2h, k, i, l).

### Permeability and separation performance of rGO–TA and rGO–TH membranes

**Fig. 3:**
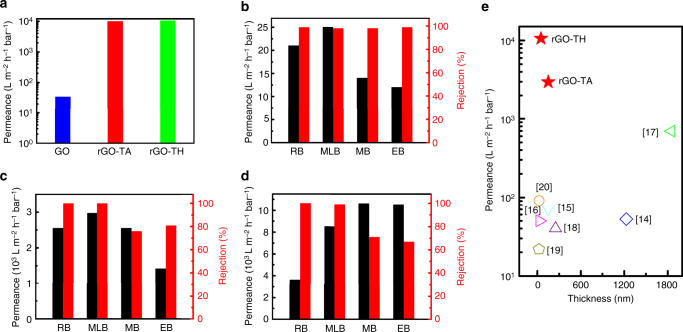
Permeability and separation performance of GO-based membranes. **a** DI water permeance of GO, rGO–TH, and rGO–TA membrane at a transmembrane pressure of 1.0 bar. **b**–**d** Permeance and rejection of organic dyes (50 µM) of GO (**b**), rGO–TA (**c**), and rGO–TH (**d**) membranes at a transmembrane pressure of 1.0 bar. GO membrane, 100 nm thick; rGO–TA membrane, 150 nm thick; rGO–TH membrane, 60 nm thick. **e** Permeance comparison of rGO–TH and rGO–TA membranes with the GO-based membranes reported in the literature^14–20^. Here, the permeance of rGO–TH and rGO–TA membranes is the maximum values that are obtained in the dye separation experiments

We then evaluated the permeability of our GO-based membranes with thicknesses ranging from 60 to 1500 nm (Fig. 3). The 100-nm-thick GO membrane shows a water permeance of 35 ± 5 L m^−2^ h^−1^ bar^−1^ (Fig. 3a), which is consistent with those reported with similar thickness^14^. This gives concrete validation for our measurement methodology. Surprisingly, the water permeance of rGO–TH membrane with a thickness of ~60 nm can reach 10,720 ± 30 L m^−2^ h^−1^ bar^−1^ (Fig. 3a). As shown in Supplementary Fig. 4, the water permeance greatly decreases with an exponential trend as the membrane thickness increases, which is very common for all the separation membranes due to the increase in mass transfer resistance. However, even when the rGO–TH membranes increase to 150 nm thickness, they still show a very high water permeance of 5000 L m^−2^ h^−1^ bar^−1^, which is more than 100 times higher than the 100-nm-thick GO membranes. More surprisingly, the rGO–TA membrane with a thickness of 150 nm shows a similar water permeance (10,191 ± 30 L m^−2^ h^−1^ bar^−1^) with the rGO–TH membrane with a thickness of 60 nm (Fig. 3a), indicating better permeability of rGO–TA membranes. This is because of the larger interlayer distance of expanded rGO membrane domains in rGO–TA membranes (~0.99 nm) than that of rGO–TH membranes (~0.85 nm) (Fig. 1i). When the thickness is decreased to 75 nm, the rGO–TA membranes show a higher water permeance (~11,600 ± 100 L m^−2^ h^−1^ bar^−1^) than the 60-nm-thick rGO–TH membranes.

To further demonstrate the advantages of our rGO–TA and rGO–TH membranes over the GO membranes and reported membranes, we measured their separation performance through pressure-driven filtration using different organic dyes as model compounds, including RB, MLB, methyl blue (MB), and evans blue (EB). GO is known as a very strong adsorbent for many organic substances, including dyes^16^. In our studies, therefore, we have intentionally excluded the adsorption effect of GO sheets by stabilizing each rejection experiment prior to the collection of permeate, feed, and retentate samples for rejection analysis (Supplementary Fig. 5), which ensures correct assessment on the separation performance.

Figure 3b–d and Table 1 summarize the separation performances of our GO-based membranes for various dyes. It can be found that the rGO–TA and rGO–TH membranes show superior permeability and high separation efficiency. The permeance of rGO–TA and rGO–TH membranes is over 100 times higher than that of GO membranes for all the dyes. Because of different charge states, the rGO–TA and rGO–TH membranes show a bit lower rejection to negatively charged MB and EB but a bit higher rejection to positively charged RB and MLB than the GO membranes. For instance, the rGO–TH membranes show a rejection of 100% and permeance of 8526 ± 30 L m^−2^ h^−1^ bar^−1^ for MLB and a rejection of 71 ± 5% and permeance of 10,602 ± 30 L m^−2^ h^−1^ bar^−1^ for MB (Fig. 3d). Since the rGO–TA and rGO–TH membranes are negatively charged at neutral pH, positively charged molecules can be easily taken up by the membranes via electrostatic interactions. As a result, the nanochannels could be partially blocked, leading to a high rejection for cationic dyes^17^. As shown in Tables 1 and 2, it is worth pointing out that the rGO–TA and rGO–TH membranes exhibit a much better balance of permeability and rejection than the reported GO-based membranes^14–20^. In particular, the permeance is 10–1000 times higher than those of the previously reported GO-based membranes^14–20^ (Fig. 3e and Table 2). Supplementary Movies 1, 2 and Supplementary Fig. 6 clearly demonstrate the superior permeability and high separation efficiency of rGO–TH membranes. Such membranes should have a great potential for industrial water treatments.

**Table 1:** Separation performance of GO-based membranes for organic dyes

Dyes	MW (g mol^−1^)	Charge	GO		rGO–TA		rGO–TH		rGO–GT	
			Perm. (L m^−2^ h^−1^ bar^−1^)	Rej. (%)	Perm. (L m^−2^ h^−1^ bar^−1^)	Rej. (%)	Perm. (L m^−2^ h^−1^ bar^−1^)	Rej. (%)	Perm. (L m^−2^ h^−1^ bar^−1^)	Rej. (%)
DW	18.0	N	35 ± 5	–	10,191 ± 30	–	10,720 ± 30	–	1550 ± 10	–
RB	479.0	+	21 ± 3	99 ± 1	2547 ± 20	100	3612 ± 20	100	1529 ± 10	100
MLB	373.0	+	25 ± 3	98 ± 1	2972 ± 20	100	8526 ± 30	99 ± 1	99 ± 1	100
MB	799.8	−	14 ± 3	98 ± 2	2547 ± 20	76 ± 5	10,602 ± 30	71 ± 5	189 ± 5	100
EB	960.0	−	12 ± 3	99 ± 1	1415 ± 20	81 ± 5	10,520 ± 30	67 ± 5	369 ± 5	81 ± 5

MW molecular weight; Perm. permeance; Rej., rejection; DW deionized water; RB rhodamine B; MLB methylene blue; MB methyl blue; EB evans blue

**Table 2:** Benchmarking of graphene-based membranes for organic dyes separation

Membrane	Preparation method	Thickness	Dyes	Rejection (%)	Permeance (L m^−2^ h^−1^ bar^−1^)	Reference
rGO + CNT	Vacuum filtration	1230 nm	MB/ AO7/ RB	98 ± 2	52.7	14
GO	Shear aligned	150 ± 15 nm	Various dyes	95 ± 5	71 ± 5	15
TMC cross-linked GO	Layer-by-layer	3–30 nm	MB	46−66	8–27.6	16
			RWT	93−95		
NSC-GO membranes	Vacuum filtration	1.85–2.17 µm	RB	87 ± 3	279	17
			EB	83 ± 1	573	
			MO	96	3.3	
CCG	Vacuum filtration	250 nm	DY	67	40	18
uGNMs	Vacuum filtration	53 nm	MB	99.8	3.2	19
		22 nm	DR 81	99.9	21.81	
HPEI/S-rGO	Vacuum filtration	25 nm	BF	86.5	97.5	20
			MB	85.4	98.6	
			EB	100	85.2	

CNTs carbon nanotubes; TMC 1,3,5-benzenetricarbonyl trichloride; NSC-GO, nanostrand-channeled graphene oxide; CCG chemically converted graphene; uGNMs ultrathin graphene nanofiltration membranes; HPEI/S-rGO, hyperbranched poly(ethylene imine)/solvent solvated reduced graphene oxide; RB rhodamine B; MB methyl blue; AO7 acid orange 7; RWT rhodamine–WT; EB evans blue; MO methyl orange; DY direct yellow; DR 81 direct red 81; MLB methylene blue; BF basic fuchsin

We then investigated the influence of the membrane thickness and the content of TA/TH molecules on the separation performance of GO-based membranes. As shown in Supplementary Table 2, although the water permeance decreases, the dye rejection increases with an increase in membrane thickness. The 200-nm-thick rGO–TH membranes show over 10% increase in MB and EB rejection compared to the 60-nm-thick membranes and still a very high water permeance that is far better than the GO membranes and reported GO-based membranes. Even for the 800-nm-thick membranes, they still show a water permeance that is better than the most reported GO-based membranes but nearly 100% rejection for MB and EB. As shown in Supplementary Tables 3 and 4, as the weight ratio of TA or TH to GO sheets increases, the membranes exhibit increased rejection for organic dyes but decreased water permeance. This confirms that the TA and TH molecules not only serve as spacer to provide a large interlayer distance to facilitate water transport but also as molecular sieves to block the dyes. It is worth noting that the water permeance is still much higher than those reported even when the weight ratio reaches 4. For instance, the rGO–TH (1:4) membranes show water permeance of 2370 ± 30 L m^−2^ h^−1^ bar^−1^ and EB rejection of 98 ± 1%. These results suggest that the separation performance of the rGO–TA and rGO–TH membranes can be further improved by optimizing the thickness and the content of TA or TH of the membranes.

### Synthesis, stability, and separation performance of rGO–GT membranes

**Fig. 4:**
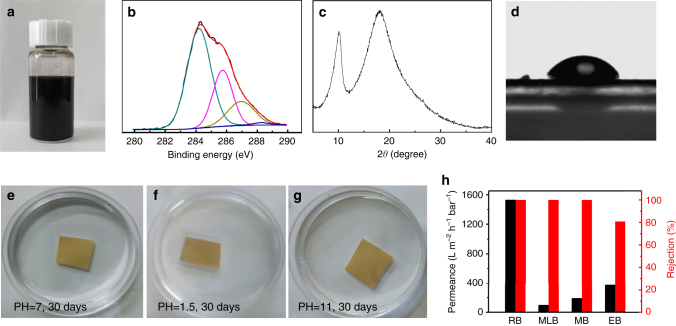
Structure and performance of rGO–GT membranes. **a** rGO–GT dispersion. **b** C 1 s XPS spectra. **c** XRD pattern. **d** Photo of a water droplet on rGO–GT membrane surface. **e**–**g** Stability in water (**e**), acid solution (**f**), and basic solution (**g**). **h** Permeance and rejection of organic dyes (1000 ppm) through a ~500-nm-thick rGO–GT membrane at a transmembrane pressure of 1.0 bar

As we know, GT extractive includes 30‒40% polyphenols (Catechins/TA) and 4‒6% amino acids (i.e., TH, glutamic acid, aspartic acid)^33,34^. To show the universality of our method, we fabricated rGO–GT membranes with GO and GT extractive. The rGO–GT membranes show similar structural features with the rGO–TA and rGO–TH membranes. As shown in Fig. 4a, b, the GO sheets are reduced by GT and the rGO–GT membrane shows an O/C atomic ratio of 0.44. Moreover, the membrane also has an enlarged interlayer distance of 0.87 nm (XRD peak at ~10.1°) (Fig. 4c), is hydrophilic (contact angle ~45°, Fig. 4d), and has a small swelling (ESR, 1.91 ± 0.21 in water) and very good stability in aqueous solutions with different pH values (Fig. 4e–g). As a result, the rGO–GT membranes also show much better separation performance than our GO membranes and those reported (Tables 1 and 2). For instance, the 500-nm-thick membranes show rejection of 100% for RB dye and high permeance of 1529 ± 10 L m^−2^ h^−1^ bar^−1^ (Fig. 4h). The big difference in XRD patterns between rGO–TA/TH and rGO–GT membranes might be due to the presence of other molecules beyond TA and TH in GT (over 50%) such as caffeine, theobromine, and theophylline^33,34^.

## Discussion

We have fabricated highly stable GO-based membranes with superior permeability and good separation efficiency by using GO sheets and TA/TH molecules. It is found that TA and TH play four roles: reduce the GO sheets to increase pristine graphitic regions as a reducing agent, link the neighboring rGO sheets as a cross-linker, enlarge the interlayer distance between rGO sheets as a spacer, and block the solutes together with the stacked rGO sheets. These membranes show water permeance over 10,000 L m^−2^ h^-1^ bar^−1^, which is 10–1000 times higher than those of the reported GO-based membranes and commercial membranes. They also show high separation efficiency to most dyes, such as nearly 100% rejection to RB and MLB. Moreover, these membranes are very stable in various aqueous solutions without any damage or delamination for months.

These findings provide a general strategy and valuable information to design 2D nanofluidic channels of GO-based membranes for selective molecular and ionic transport by using small organic molecules. As an example, we demonstrate the universality of this method to fabricate high-performance dye separation membranes by simply using GT extractive. We anticipate that this method can also be used for other 2D materials, and the resultant membranes not only have a great potential in water treatments, but also opens up new possibilities to study wet-chemical reactions confined in the few nanometers of space between layers.

## Methods

### Synthesis of GO sheets

The GO sheets were prepared by using the modified Hummers method^29^. First, 3.0 g of graphite powder (325 mesh, Sigma Aldrich Co., Ltd.), 1.5 g of NaNO_3_, and 96 mL of concentrated H_2_SO_4_ were mixed together in ice bath with constant magnetic stirring. Then, 9.0 g of KMnO_4_ was slowly added to the above reaction mixture at below 20 °C. The obtained mixture was stirred below 20 °C for 90 min and then at 35 °C for another 2 h. After that, DI water (138 mL) was added dropwise into the reaction mixture to avoid overheating. Then, 420 mL of DI water was added together with 3 mL of H_2_O_2_ (30%) to obtain a graphite oxide suspension. The product was repeatedly washed with 3% HCl aqueous solution continuously and dialysis up to 3 days to remove metallic contamination until the pH = 7. Tip-sonication (135 W, 60 min) was then used to exfoliate the graphite oxide into GO suspension. Finally, the suspension was successively centrifuged at 3000 and 10,000 rpm for 30 min to remove thick multilayer flakes and small pieces, respectively. The obtained GO dispersion was further dried and used for the fabrication of membranes.

### Synthesis of GO, rGO–TA, and rGO–TH membranes

First, the coating solution (2 mg mL^−1^) was prepared by dissolving 0.16 g of dopamine hydrochloride into 80 mL of 0.01 M Tris buffer at pH = 8.5. The PES membranes (pore size of 0.45 μm, measured water permeance around 34,000 ± 500 L m^−2^ h^−1^ bar^−1^, Tianjin Branch Billion Lung Experimental Equipment Co. Ltd.) were then immersed in this solution at room temperature for 2 h. During this period, dopamine underwent self-polymerization and formed an adhesive layer on the substrate to anchor GO sheets. After that, the substrates were rinsed in DI water and dried in an oven at 60 °C. To prepare the rGO–TA and rGO–TH membranes, 0.25 mg GO powder and different amounts of TA/TH (0.25 mg, 0.5 mg, 0.75 mg, and 1.0 mg) were fully dispersed/dissolved in 40 mL of DI water and sonicated for 1 h to make sure complete mixing between TA/TH and GO and then further stirred overnight at 70 °C for reaction. The dispersion was then filtrated through the modified PES membrane in a dead-end filtration cell. For comparison, GO membranes were prepared following the same procedure but without the addition of TA/TH.

### Synthesis of rGO–GT membranes

1.5 g of green tea powder was dissolved in 40.0 mL of water at 80 °C for 24 h. The extract was filtered through paper filter (Grade 1, Whatman) by Büchner filtration. Then, 1.5 g of GO sheets was added into the extract and stirred for 24 h at room temperature. The as-prepared dispersion (~10 mL) was diluted into 40 mL with water, followed by ultrasonication for 30 min. The resulting dispersions were vacuum filtered on PES membrane (pore size, 0.45 µm). After filtration, the samples were dried at room temperature overnight before use.

### Membranes characterization

The morphologies and structures of GO sheets and GO-based membranes were characterized by scanning electron microscope (SEM, Nova NanoSEM 430, 15 kV/10 kV/5 kV), and the thicknesses were measured by Bruker DektakXT Stylus Profiler (Germany). The chemical compositions of membranes were characterized by XPS on ESCALAB250 (150 W, spot size 500 µm) using Al Kα radiation; all spectra were calibrated to the binding energy of adventitious carbon (284.6 eV). XRD patterns were acquired with an XRD diffractometer (D-MAX/2400) using Cu Kα radiation (λ = 0.154 nm). Hydrophilicity of the membrane surfaces was evaluated by a contact angle goniometer. UV–Vis spectra for the dyes were measured on a UV–Vis–NIR spectrophotometer (Varian Carry 5000). AFM (Bruker, Multimode 8) was used to characterize the thickness of GO sheets. FTIR spectra were measured on Bruker Tensor 27 to identify the cross-linking between TA/TH and rGO sheets.

### Water permeability and dyes rejection of the membranes

The water permeance and dyes rejection of the membranes were measured on a home-made dead-end vacuum filtration device with an effective area of 14.51 cm^2^ under a pressure difference of 1.0 bar at room temperature. The volume of feed solution was 250 mL for GO, rGO–TA and rGO–TH membranes and 1000 mL for rGO–GT membranes. First DI water was used to test the pure water permeance of the membrane, and then different organic dyes were used to test the rejection performance. For the GO and rGO–GT membranes, it usually takes a long time to get a stable water permeance. Therefore, the water permeance was recorded every 10 min for GO and rGO–GT membranes until it became steady and the stable value was recorded as the permeance. For the rGO–TA and rGO–TH membranes, as shown in Supplementary Movies 1 and 2, the permeance is very high and becomes steady very quickly. Therefore, we directly recorded the permeance of rGO–TA and rGO–TH membranes when the feed pressure reached 1.0 bar until all the solutions were completely filtered out. The permeance *J* (L m^−2^ h^−1^ bar^−1^) and rejection *R* (%) were calculated according to Eq. (1) and Eq. (2) respectively:

1 $$J = \frac{V}{{A\Delta tP}}$$

2 $$R = 1 - \frac{{C_{\mathrm{p}}}}{{C_{\mathrm{f}}}} \times 100{\mathrm{\% }}$$

where *V* (L) is the volume of permeated water, *A* (m^2^) is the effective membrane area, *Δt* (h) is the permeate time, *P* (1.0 bar) is the pressure difference, and *C*_p_ and *C*_f_ are the concentration of permeate and feed solution, respectively.

### Stability tests

The as-prepared membranes were cut into 1 × 1 cm^2^ pieces and then statically immersed in pure water (pH = 7 ± 0.2), HCl aqueous solution (pH = 1.5), and NaOH aqueous solution (pH = 11), respectively, at room temperature. The stabilities of the membranes in different solutions were recorded after the membranes have been immersed for a certain time.

### Determination of the degree of swelling

The dried membranes were immersed into DI water for 24 h at 30 °C and then dried in vacuum. The degree of swelling (*D*) was calculated by

3 $$D = \frac{{W_2 - W_1}}{{W_1}}$$

where *W*_1_ and *W*_2_ are the weights of the original and treated membranes, respectively.

### Data availability

The data that support the findings of this study are available from the corresponding author upon request.

## Electronic supplementary material

Supplementary Information

Peer Review Report

Description of Additional Supplementary Files

Supplementary Movie 1

Supplementary Movie 2

## References

[1] Mohammad AW (2015). Nanofiltration membranes review: recent advances and future prospects. Desalination.

[2] Dijkstra HP, Van Klink GPM, Van Koten G (2002). The use of ultra- and nanofiltration techniques in homogeneous catalyst recycling. Acc. Chem. Res..

[3] Dioos BML, Vankelecom IFJ, Jacobs PA (2006). Aspects of immobilisation of catalysts on polymeric supports. Adv. Synth. Catal..

[4] Stankovich S (2007). Synthesis of graphene-based nanosheets via chemical reduction of exfoliated graphite oxide. Carbon N. Y..

[5] Dreyer DR, Park S, Bielawski CW, Ruoff RS (2010). The chemistry of graphene oxide. Chem. Soc. Rev..

[6] Pei SF, Cheng HM (2012). The reduction of graphene oxide. Carbon N. Y..

[7] Nair R (2012). Unimpeded permeation of water through helium-leak–tight graphene-based membranes. Science.

[8] Joshi R (2014). Precise and ultrafast molecular sieving through graphene oxide membranes. Science.

[9] Liu G, Jin W, Xu N (2015). Graphene-based membranes. Chem. Soc. Rev..

[10] Abraham J (2017). Tunable sieving of ions using graphene oxide membranes. Nat. Nanotechnol..

[11] Sun P, Wang K, Zhu H (2016). Recent developments in graphene-based membranes: structure, mass-transport mechanism and potential applications. Adv. Mater..

[12] Huang L, Zhang M, Li C, Shi G (2015). Graphene-based membranes for molecular separation. J. Phys. Chem. Lett..

[13] Perreault F, De Faria AF, Elimelech M (2015). Environmental applications of graphene-based nanomaterials. Chem. Soc. Rev..

[14] Goh K (2015). All-carbon nanoarchitectures as high-performance separation membranes with superior stability. Adv. Funct. Mater..

[15] Akbari A (2016). Large-area graphene-based nanofiltration membranes by shear alignment of discotic nematic liquid crystals of graphene oxide. Nat. Commum..

[16] Hu M, Mi B (2013). Enabling graphene oxide nanosheets as water separation membranes. Environ. Sci. Technol..

[17] Huang H (2013). Ultrafast viscous water flow through nanostrand-channelled graphene oxide membranes. Nat. Commun..

[18] Qiu L (2011). Controllable corrugation of chemically converted graphene sheets in water and potential application for nanofiltration. Chem. Commun..

[19] Han Y, Xu Z, Gao C (2013). Ultrathin graphene nanofiltration membrane for water purification. Adv. Funct. Mater..

[20] Huang L (2016). Reduced graphene oxide membranes for ultrafast organic solvent nanofiltration. Adv. Mater..

[21] Dikin DA (2007). Preparation and characterization of graphene oxide paper. Nature.

[22] Chen CM (2009). Self-assembled free-standing graphite oxide membrane. Adv. Mater..

[23] Zambare RS, Dhopte KB, Patwardhan AV, Nemade PR (2017). Polyamine functionalized graphene oxide polysulfone mixed matrix membranes with improved hydrophilicity and anti-fouling properties. Desalination.

[24] Zhang Y, Zhang S, Chung T (2015). Nanometric graphene oxide framework membranes with enhanced heavy metal removal via nanofiltration. Environ. Sci. Technol..

[25] Yeh C (2015). On the origin of the stability of graphene oxide membranes in water. Nat. Chem..

[26] Park SJ (2008). Graphene oxide papers modified by divalent ions—enhancing mechanical properties via chemical cross-linking. ACS Nano.

[27] Liu H, Wang H, Zhang X (2015). Facile fabrication of freestanding ultrathin reduced graphene oxide membranes for water purification. Adv. Mater..

[28] Lee A, Elam JW, Darling SB (2016). Membrane materials for water purification: design, development, and application. Environ. Sci.—Water Res. Technol..

[29] Hummers WS, Offeman RE (1958). Preparation of graphitic oxide. J. Am. Chem. Soc..

[30] Pei SF (2010). Direct reduction of graphene oxide films into highly conductive and flexible graphene films by hydrohalic acids. Carbon N. Y..

[31] Simons, W. W. *Sadtler Handbook of Infrared Spectra* Vol.11, 21A (Sadtler Research Laboratories, Philadelphia, 1978).

[32] Max JJ, Chapados C (2004). Infrared spectroscopy of aqueous carboxylic acids: comparison between different acids and their salts. J. Phys. Chem. A.

[33] Puligundla P (2017). Nanotechnological approaches to enhance the bioavailability and therapeutic efficacy of green tea polyphenols. J. Funct. Foods.

[34] Sharangi AB (2009). Medicinal and therapeutic potentialities of tea (camellia sinensis L.)—a review. Food Res. Int..

