# Supplementary material for: Highly stable graphene-oxide-based membranes with superior permeability
Source: Nat Commun. 2018 Apr 16;9:1486. doi: 10.1038/s41467-018-03919-0 (PMC5902455; doi:10.1038/s41467-018-03919-0)
Supplement: Supplementary file 1 — Supplementary Information [file 41467_2018_3919_MOESM1_ESM.pdf]

Highly stable graphene-oxide-based membranes with superior permeability

Thebo et al.

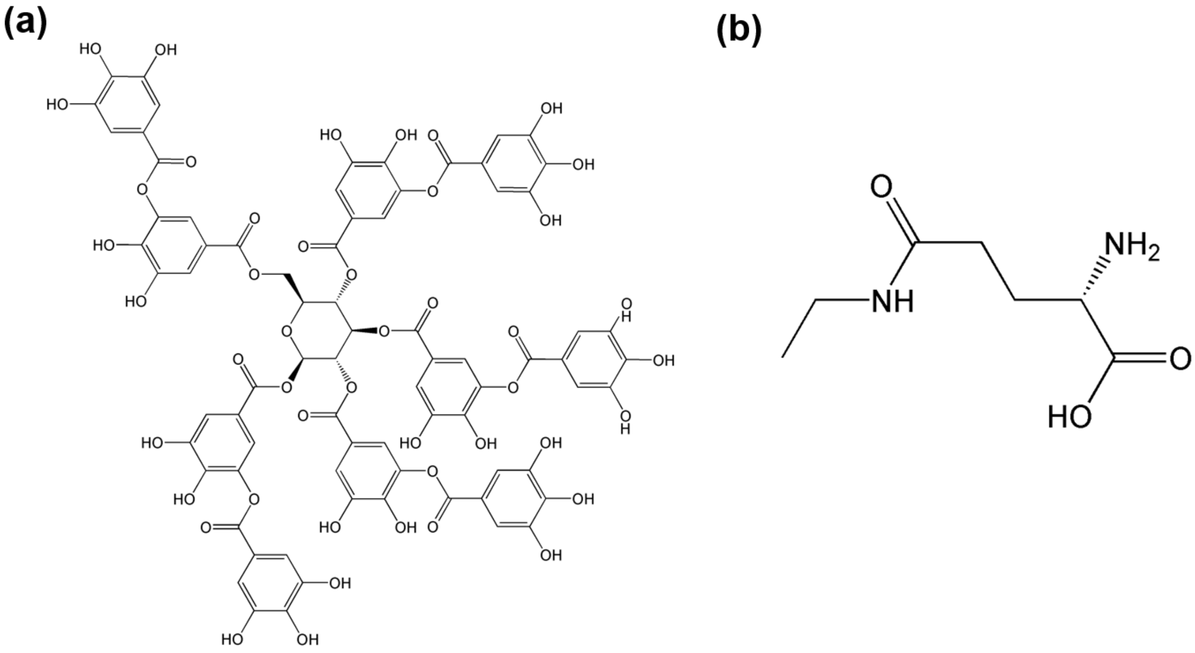

Supplementary Figure 1 Molecular structure of (**a**) tannic acid and (**b**) theanine amino acid.

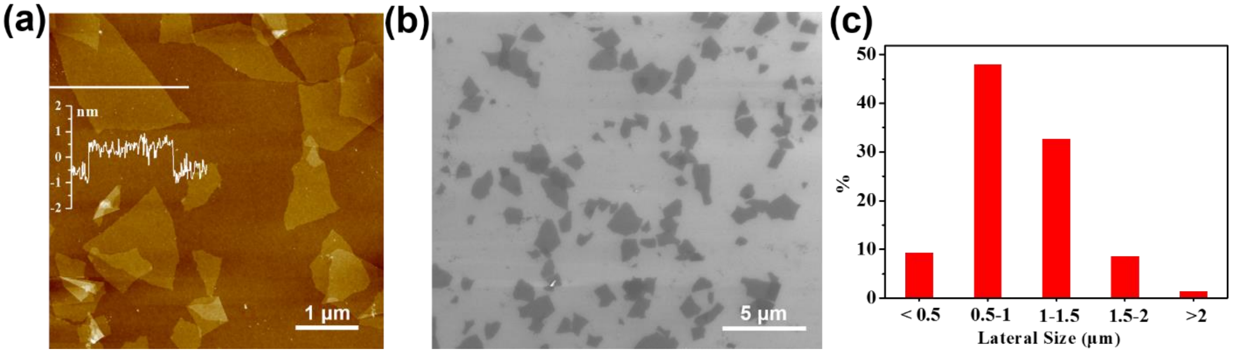

Supplementary Figure 2 AFM (**a**), SEM (**b**), and lateral size distribution (**c**) of GO sheets.

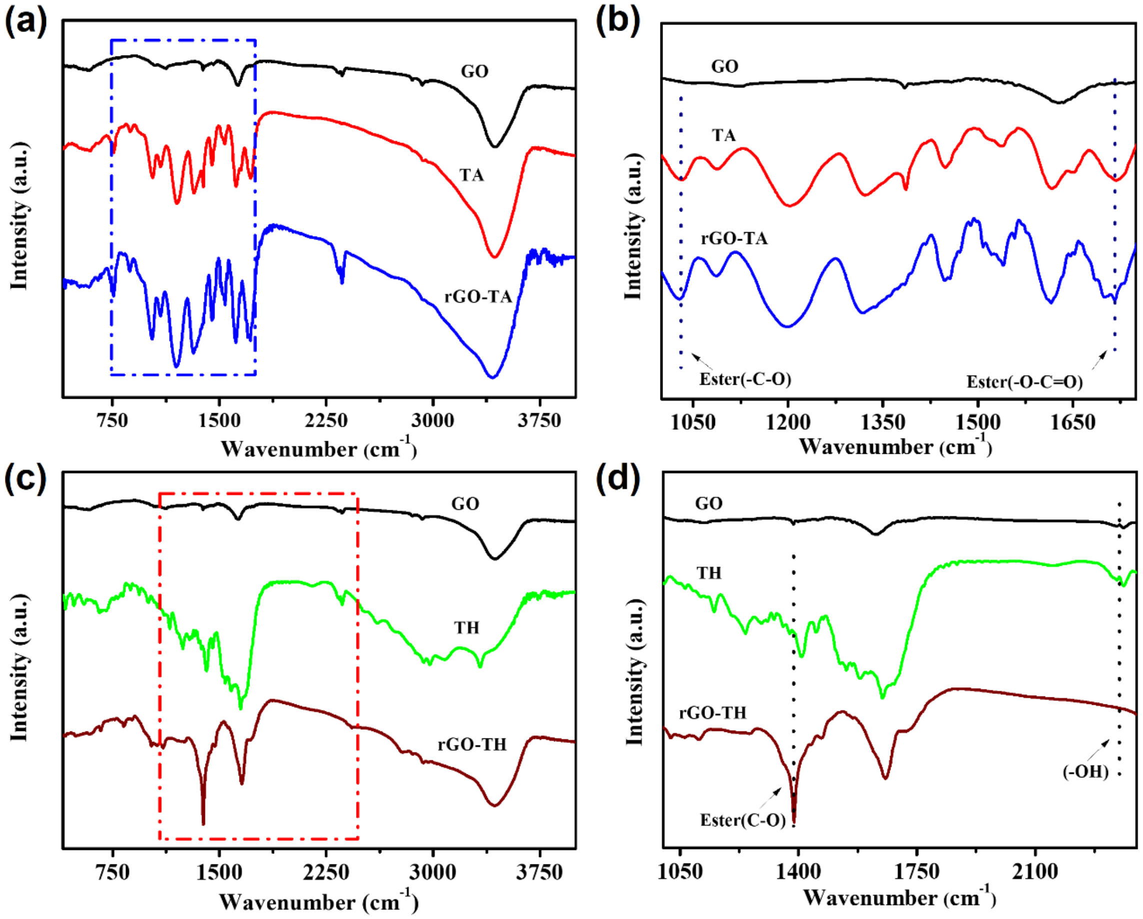

Supplementary Figure 3 (**a**) FTIR spectra of GO membrane, TA, and rGO-TA membrane. (**b**) Zoom in of the area indicated by blue square in **a**. (**c**) FTIR spectra of GO membrane, TH, and rGO-TH membrane. (**d**) Zoom in of the area indicated by red square in **c**.

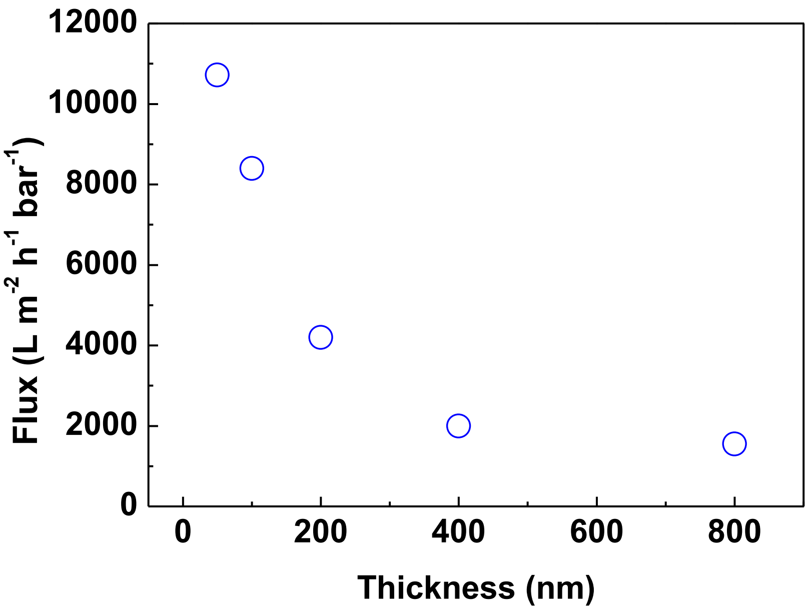

Supplementary Figure 4 The permeance of rGO-TH membranes as a function of thickness.

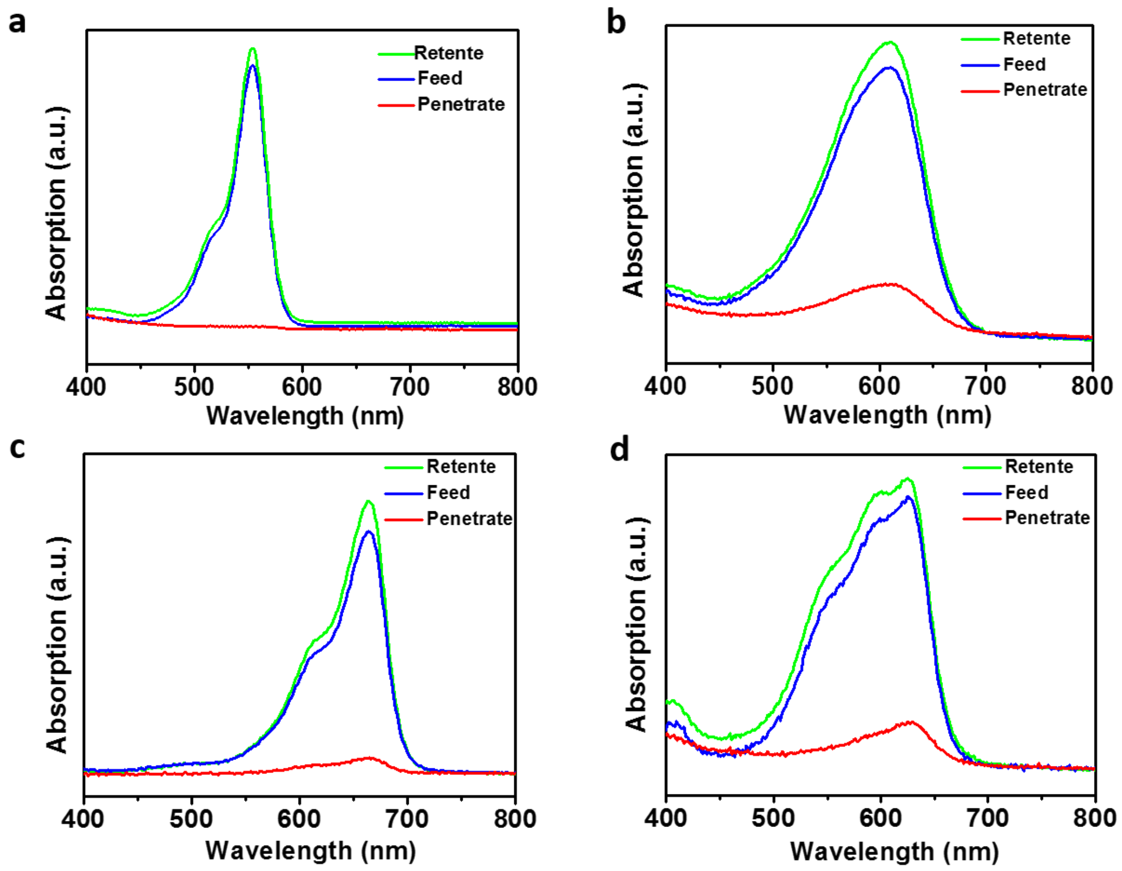

Supplementary Figure 5 UV-vis absorption spectra of the feed, the permeate and the retentate of (**a**) RB, (**b**) EB, (**c**) MLB and (**d**) MB solution after filtration by rGO-TH membrane (60 nm).

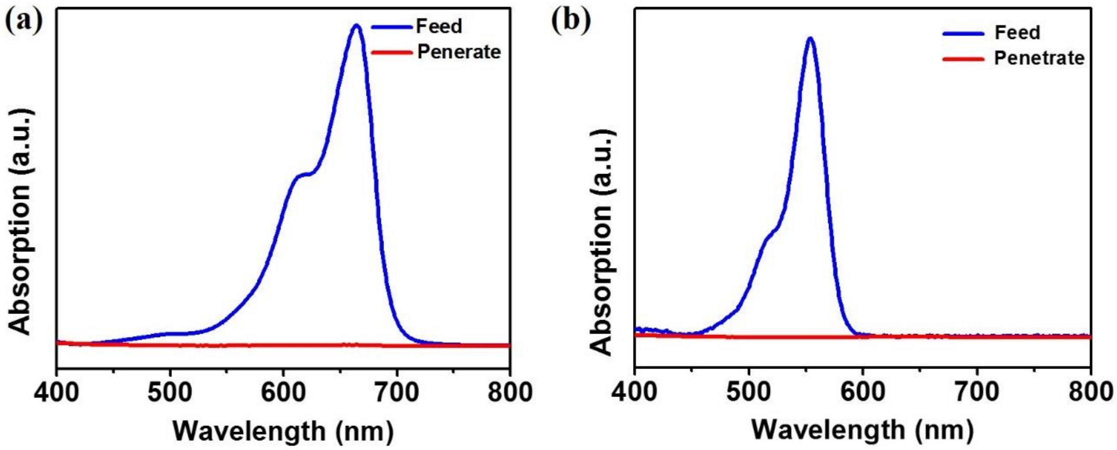

Supplementary Figure 6 UV-vis absorption spectra of the feed and permeate of (**a**) MLB, and (**b**) RB solution after filtration through ~60-nm-thick rGO-TH membrane.

Supplementary Table 1 The equilibrium weight swelling ratio (ESR) of GO, rGO-TA (50 wt.%), rGO-TH (50 wt.%), and rGO-GT (50 wt.%) membranes in water.

Membranes	ESR
GO	2.21 ± 0.11
rGO-TA	1.33 ± 0.05
rGO-TH	1.78 ± 0.15
rGO-GT	1.91 ± 0.21

Supplementary Table 2 The separation performance of rGO-TH membranes with different thickness.

Membrane Thickness (nm)	MB		EB		RB	
	Permeance (LMBH)	Rejection (%)	Permeance (LMBH)	Rejection (%)	Permeance (LMBH)	Rejection (%)
60	10,602±30	71±5	10,520±30	67±5	3,612±20	99±1
100	7,620±30	75±2	7,860±30	71±2	2,045±50	100
200	4,550±30	83±3	5,120±30	80±3	1,520±20	100
400	780 ±50	95±2	950±30	92±2	645±20	100
800	275 ±50	99±1	420±30	98±1	230±20	100

Supplementary Table 3 The separation performance of rGO-TA membranes with different weight ratio of TA to GO.

-	rGO-TA (1:1) (150 nm)		rGO-TA (1:2) (145±5 nm)		rGO-TA (1:3) (150±5 nm)		rGO-TA (1:4) (150 ±5 nm)	
	Perm.	Rej.	Perm.	Rej.	Perm.	Rej.	Perm.	Rej.
DW	10,191±30	---	8,280±50	----	4,445±50	----	2,570±50	----
RB	2,547±20	100	2,075±50	100	1,545±30	100	1,230±30	100
EB	1,415±20	81±5	1,310±30	92±1	830±30	99±1	680±30	99±1
MLB	2,972±20	100	2,150±50	100	1,850±30	100	1,320±20	100

Supplementary Table 4 The separation performance of rGO-TH membranes with different weight ratio of TH to GO.

-	rGO-TH (1:1) (60 nm)		rGO-TH (1:2) (65±5 nm)		rGO-TH (1:3) (65±5 nm)		rGO-TH (1:4) (65 ±5 nm)	
	Perm.	Rej.	Perm.	Rej.	Perm.	Rej.	Perm.	Rej.
DW	10,720±30	---	8,530±50	--	6,250±30	---	4,570±30	---
RB	3,612±20	100	2,520±50	100	1,375±30	100	945±30	100
MLB	8,526±30	99±1	6,250±50	100	3,125±30	100	1,250±30	100
EB	10,520±30	67±5	6,730±30	80±3	3,370±30	95±2	2,370±30	98±1
