# Supplementary material for: Highly stable graphene-oxide-based membranes with superior permeability
Source: Nat Commun. 2018 Apr 16;9:1486. doi: 10.1038/s41467-018-03919-0 (PMC5902455; doi:10.1038/s41467-018-03919-0)
Supplement: Supplementary file 3 — Description of Additional Supplementary Files [file 41467_2018_3919_MOESM3_ESM.pdf]

Description of Additional Supplementary Files

File name: Supplementary Movie 1
Description: Filtration of MLB through rGO-TH membrane. 250 mL MLB (50 μM) solution is filtered through the ~60-nm-thick rGO-TH membrane using vacuum filtration set-up. The effective filtration area is 11.34 cm^2, and the feed pressure is 1.0 bar. Note that the 250 mL MLB solution is filtered out by 94 seconds and the MLB is totally rejected as confirmed by UV-vis absorption spectra (Supplementary Fig. 6a).

File name: Supplementary Movie 2
Description: Filtration of RB through rGO-TH membrane. 250 mL RB solution (50 μM) is filtered through a ~60-nm-thick rGO-TH membrane using vacuum filtration set-up. The effective filtration area is 11.34 cm^2, and the feed pressure is 1.0 bar. Note that the 250 mL RB solution is filtered out by 230 seconds and the RB is totally rejected as confirmed by UV-vis absorption spectra (Supplementary Fig. 6b).
