# Supplementary material for: Highly stable graphene-oxide-based membranes with superior permeability
Source: Nat Commun. 2018 Apr 16;9:1486. doi: 10.1038/s41467-018-03919-0 (PMC5902455; doi:10.1038/s41467-018-03919-0)
Supplement: Supplementary file 2 — Peer Review Report [file 41467_2018_3919_MOESM2_ESM.pdf]

Reviewers' comments:

Reviewer #1 (Remarks to the Author):

Authors report using the main ingredients of green tea to reduce and cross-link GO membranes. The resulting membrane shows record-high water permeance and good selectivity to dyes. The idea is novel and interesting, however, there are several comments need to be addressed before the consideration for possible publication.

1. the record-high water permeance:
-- experiment part. How about the water permeance for the PES substrate used in this study? This might justify the ultrahigh water permeance in this study.
Adopt data from Millipore, who is a main provider for porous polymeric substrate, the PES membrane gives a flow rate of 40 mL/min cm2 at 2 bar, 25 °C (https://www.merckmillipore.com/GB/en/product/Millipore-Express-P LUS-Membrane-Filters,MM_NF-C2997?ReferrerURL=https%3A%2F%2Fwww.go ogle.co.uk%2F&bd=1#specifications), that is 12,000 L m-2 h-1 bar-1(LMBH). The reported permeance of 10,720 ± 30 LMBH for GO-TH membrane suggests the water permeance of GO membrane is equal or might be higher than the porous PES substrate. That is really quite surprising.
--60 nm thin GO-TH and 150 nm thin GO-TA membrane show similar water permeance. At least a primary explanation on why GO-TH and GO-TA show different thickness dependent water permeance is needed. Furthermore, does this mean thinner GO-TA membrane, e.g. 60 nm, could show even higher water permeance?
--Authors explains the ultrafast water permeation by alternating structure of GO-TH and GO-TA membranes, in which, oxidized regions attract water and graphitic regions allow slip transport. However, this mechanism applies to pristine GO membrane as well(Science 2012, 335, 442-4.), therefore, this explanation should not be enough to explain 2-3 orders of magnitude higher water permeance than GO membrane.
--A minor comment on terminology, see J. Membr. Sci. 2010, 348, 346-352., Flux should be in a unit of L m-2 h-1, permeance should be in a unit of L m-2 h-1 bar-1, permeability should be the permeance normalized with thickness.

2. Cross-linking
-- It is claimed that TA and TH molecules are cross-linked to GO sheets, What is the possible interaction between TA, TH and GO sheets to achieve cross-linking?
--Could authors provide more solid evidences on cross-linking effect? as in the study, TA and TH are used to reduce GO membrane as well, as a consequence of removal of functional groups, one can attribute the water stability to reduction effect rather than cross-linking(Fig. 2).
-- weight ratio, at least a ratio range between TA,TH to GO should be given for experiment reproduction by potential readers.

3.Characterization
--Raman is mentioned in the experiment session, but the Raman spectra are not seen in either main text or SI.
--For the XRD, the reduction peaks in Fig. 1i and Fig. 4c are quite different both in peak position and intensity. As TA, TH are main ingredients of green tea, why their XRD spectra exhibit such huge differences?
--Two typos, main text, page 15, line 250, "were s prepared",
and SI, caption for SI Fig. 1, "theanine amino acidic"

Reviewer #2 (Remarks to the Author):

Tailoring the structure of graphene oxide (GO) membranes is essentially important for various applications. Recently, GO membranes have been widely demonstrated to have a great potential for water purification, desalination and molecular separation. However, these membranes suffer from a low stability and are easily damaged in aqueous solutions. How to further improve the water permeability without sacrificing the separation efficiency is also very challenging. In this manuscript, the authors fabricated a unique GO-based membranes by using theanine amino acid, tannic acid, and even green tea extractives as cross-linker. Very interestingly, these membranes show alternating GO membrane-like and reduced GO membrane-like domains, which enable excellent stability and superior permeability simultaneously. They show no damage in various aqueous solutions even after months, and water permeation of 10 – 1,000 times higher than those of the reported GO-based membranes and commercial membranes, together with good separation efficiency.

This work is of high novelty and urgent important for not only water treatment but also many solution-phase applications of GO membranes. It also provides valuable information to design rationally 2D nanofluidic channels for selective molecular and ionic transport, which I believe will attract broad interest of 2D material community. Therefore, it can be accepted for publication after minor revision.

1) Please give more detailed information of the GO sheets used for the fabrication of membranes, such as the lateral size, number of layers, etc.

2) The authors showed that the water permeability of the membranes strongly depends on their thickness. Have the authors checked the effect of the membrane thickness on the separation efficiency to dyes? Is it possible to further improve the separation efficiency without a great decrease in water permeability by optimizing the thickness of the membranes?

3) The authors are suggested to give more discussions about the origin of the superior water permeability and the different separation efficiency to different dyes of the cross-linked GO-based membranes.

4) There are words misleading in the manuscript. For example, in line 122, there is a word ‘perquisite’, do the authors mean ‘prerequisite’?

Response to Reviewers' Comments

Reviewer #1 (Remarks to the Author):

Authors report using the main ingredients of green tea to reduce and cross-link GO membranes. The resulting membrane shows record-high water permeance and good selectivity to dyes. The idea is novel and interesting, however, there are several comments need to be addressed before the consideration for possible publication.

Reply: We thank the reviewer very much for positive comments.

1. The record-high water permeance:

-- Experiment part. How about the water permeance for the PES substrate used in this study? This might justify the ultrahigh water permeance in this study. Adopt data from Millipore, who is a main provider for porous polymeric substrate, the PES membrane gives a flow rate of 40 mL/min cm^2 at 2 bar, 25 °C (https://www.merckmillipore.com/GB/en/product/Millipore-Express-PLUS-Membrane-Filters,MM_NF-C2997?ReferrerURL=https%3A%2F%2Fwww.google.co.uk%2F&bd=1#specifications), that is 12,000 L m^{-2} h^{-1} bar^{-1} (LMBH). The reported permeance of 10,720 ± 30 LMBH for GO-TH membrane suggests the water permeance of GO membrane is equal or might be higher than the porous PES substrate. That is really quite surprising.

Reply: We thank the reviewer very much for kind comments.

It is true that our GO-TH (10,720 ± 30 L m^{-2} h^{-1} bar^{-1}) and GO-TA membranes (10,191 ± 30 L m^{-2} h^{-1} bar^{-1}) have similar permeance with the PES membranes that are provided by Millipore (~12,000 L m^{-2} h^{-1} bar^{-1}). The PES membranes used in our study are purchased from Tianjin Branch Billion Lung Experimental Equipment Co. Ltd. The measured water permeance of these PES membranes is 34,000 ± 500 L m^{-2} h^{-1} bar^{-1}, which is much higher than our modified GO membranes.

We have added the detailed information of the PES membranes in the revised

manuscript.

-- 60 nm thin GO-TH and 150 nm thin GO-TA membrane show similar water permeance. At least a primary explanation on why GO-TH and GO-TA show different thickness dependent water permeance is needed. Furthermore, does this mean thinner GO-TA membrane, e.g. 60 nm, could show even higher water permeance?

Reply: We thank the reviewer very much for kind suggestion.

The size of TA molecule is larger than TH molecule (Supplementary Fig. 1). As a result, the interlayer distance of the rGO domains inserted with TA molecules in GO-TA membranes (~0.99 nm) is little bit larger than that of the rGO domains inserted with TH molecules in GO-TH membranes (~0.85 nm) (Fig. 1i). The larger interlayer distance is beneficial for the water permeation in the nanochannels. Therefore, 150-nm-thick GO-TA membrane and 60-nm-thick GO-TH membrane show similar water permeance. In order to evaluate the performance of thinner GO-TA membrane, we have fabricated GO-TA membranes with a thickness around 75 nm. These membranes show higher permeance (~11,600 ± 100 L m^{-2} h^{-1} bar^{-1}) than the 60-nm-thick GO-TH membranes (~10,720 ± 30 L m^{-2} h^{-1} bar^{-1}). Unfortunately, they show low rejection towards dyes such as RB (65 ± 3%) and EB (45 ± 3%). Therefore, 60-nm-thick GO-TH membranes are more suitable than the 75-nm-thick GO-TA membranes for dye separation application in terms of high water permeance and good rejection. The details of the water permeance and dye rejection of 75-nm-thick GO-TA membranes are listed in Table R1.

We have added the above information in the revised manuscript.

Table R1. Water permeance and dye rejection of 75-nm-thick GO-TA membranes.

Molecules	Permeance (L m^{-2} h^{-1} bar^{-1})	Rejection (%)
Water	11,580 ± 100	-
RB	5,145 ± 50	65± 3

EB	7,675 ± 50	45± 3

-- Authors explains the ultrafast water permeation by alternating structure of GO-TH and GO-TA membranes, in which, oxidized regions attract water and graphitic regions allow slip transport. However, this mechanism applies to pristine GO membrane as well (Science 2012, 335, 442-4.), therefore, this explanation should not be enough to explain 2-3 orders of magnitude higher water permeance than GO membrane.

Reply: We thank the reviewer very much for kind comment.

Pristine GO membranes have oxidized regions and graphitic regions. As reported previously (*Science* 2012, 335, 442-444 and 2014, 343, 752-754), the oxidized regions serve as spacer and graphitic regions allow slip transport. However, the graphitic regions are very limited in pristine GO membranes. Moreover, the pristine GO membranes show a single XRD peak around 11.6º as shown in our manuscript, corresponding to an interlayer distance of ~0.76 nm.

As mentioned in our manuscript, reduction and cross-linking take place simultaneously when TA and TH are used. Reduction leads to greatly increased graphitic regions. It is worth noting that both the GO-TA and GO-TH membranes show two XRD peaks. The weak peak is located at around 26.4º, which is attributed to the narrowed interlayer distance of neighboring rGO sheets that are attracted each other by π-π interaction. These rGO membrane domains are not suitable for water permeation because of the very small interlayer distance but play an important role in the stability of membranes. The much stronger XRD peak is located at ~8.9º and 10.4º for GO-TA and GO-TH membranes, respectively, corresponding to interlayer distance of 0.99 nm and 0.85 nm. This indicates that TA or TH molecules are inserted between the neighboring rGO sheets in most of the regions of GO-TA and GO-TH membranes, which act as spacer to ensure a relatively large nanochannel to facilitate water transport. Therefore, we suggest that the greatly increased graphitic regions and enlarged interlayer distance in these rGO membrane domains are responsible for the much higher water permeance than GO membranes. In addition, the covalent bonding

between TA/TH molecules and rGO sheets, as shown below, also plays an important role in the stability of the membranes in aqueous solutions.

We have added the above discussions in the revised manuscript.

-- A minor comment on terminology, see J. Membr. Sci. 2010, 348, 346-352., Flux should be in a unit of L m^{-2} h^{-1}, permeance should be in a unit of L m^{-2} h^{-1} bar^{-1}, permeability should be the permeance normalized with thickness.

Reply: We thank the reviewer very much for the good suggestion.

We have replaced flux with permeance in the revised manuscript.

2. Cross-linking

-- It is claimed that TA and TH molecules are cross-linked to GO sheets, what is the possible interaction between TA, TH and GO sheets to achieve cross-linking?

Reply: We thank the reviewer very much for kind comment.

As we know, GO contains several oxygen containing functional groups such as hydroxyl, epoxy, carbonyl, and carboxyl groups on basal plane and edges, which can act as anchoring sites to hold various cross-link reagents. As shown in Supplementary Fig. 1, TA molecules have abundant hydroxyl groups, while TH molecules contain both hydroxyl and amine functional groups.

We used Fourier Transform Infrared (FTIR) spectroscopy to characterize the GO, GO-TA and GO-TH membranes (Supplementary Fig. 3). Compared to GO membranes, GO-TA membranes show two strong new peaks at 1025 and 1718 cm^{-1}, which correspond to the stretching vibration of the –C-O-C moiety and -C=O- moiety in ester groups (-O-C=O-), respectively (*The Sadtler Handbook of Infrared Spectra,* 1978, 11, 21A). These two peaks are also observed in TA molecules but show much weaker intensity. The increased peak intensities indicate that the residual carboxyl groups of rGO sheets have covalently bonded with the hydroxyl groups of TA molecules, leading to more ester groups. Similarly, GO-TH membranes show much stronger ester C-O stretch peak at 1389 cm^{-1}. Moreover, the peak at 2357 cm^{-1}, which

corresponds to the carboxylic (-OH) stretching of -COOH in TH molecules (*J. Phys. Chem. A*, 2004, 108, 3324-3337), is absent in GO-TH membranes. These confirm that the -COOH groups of TH molecules have been covalently bonded with the residual oxygen functional groups of rGO sheets. All these results suggest the existence of strong covalent interactions between rGO sheets and TA/TH molecules. In addition, there should exist hydrogen bonding interactions between rGO sheets and TA/TH molecules and π-π interactions between rGO sheets and TA molecules.

We have added the FTIR data and the above discussions in the revised manuscript.

--Could authors provide more solid evidences on cross-linking effect? as in the study, TA and TH are used to reduce GO membrane as well, as a consequence of removal of functional groups, one can attribute the water stability to reduction effect rather than cross-linking (Fig. 2).

Reply: We thank the reviewer very much for kind suggestion.

As shown in the above reply, the rGO sheets are cross-linked with TA/TH molecules by covalent bonds. It is reasonable to expect that such strong bonding plays an important role in the water stability of the modified GO membranes. The good stability of rGO membranes in water is attributed to the narrowed interlayer distance of neighboring rGO sheets caused by π-π interactions, which has no space for permeation of water molecules (*Nat. Common.* 2014, 5, 4843). As shown in Fig. 1i, there are a great number of regions with interlayer distance larger than that of GO membranes in GO-TA and GO-TH membranes. These regions would be delaminated if only reduction effect plays role in the stability. However, these two membranes show excellent stability without visible damage or delamination being observed even after over 30 days in different aqueous solutions. Therefore, we suggest that both the cross-linking and reduction are responsible for the excellent water stability of GO-TA and GO-TH membranes.

We have made the reasons for the excellent water stability of modified GO membranes more clearly in the revised manuscript.

--Weight ratio, at least a ratio range between TA, TH to GO should be given for experiment reproduction by potential readers.

Reply: We thank the reviewer very much for kind suggestion.

The weight ratio of TA/TH molecules to GO sheets in the membranes shown in our manuscript is 1:1. In order to investigate the influence of the content of TA/TH on the separation performance of modified membranes, we have synthesized GO-TA and GO-TH membranes with different weight ratio of TA/TH to GO using 0.25 mg GO sheets and different amounts of TA/TH molecules (0.25 mg, 0.5 mg, 0.75 mg and 1.0 mg). As shown in Tables R2 and R3, as the weight ratio of cross-link reagent increases, the membranes exhibit increased rejection for organic dyes but decreased water permeance, which might due to the blocked nanochannels between rGO nanosheets by cross-link reagents. However, it is worth noting that the water permeance is still much higher than those reported even when the weight ratio of cross-link reagent to GO sheets reaches 4. This provides a way to optimize the performance of the membranes by simply changing the content of TA/TH.

We have added the data and related discussions in the revised manuscript.

Table R2. The separation performance of GO-TA membranes with different weight ratio of TA to GO.

-	GO-TA (1:1) (150 nm)		GO-TA (1:2) (145±5 nm)		GO-TA (1:3) (150±5 nm)		GO-TA (1:4) (150 ±5 nm)	
	Perm.	Rej.	Perm.	Rej.	Perm.	Rej.	Perm.	Rej.
DW	10,191±30	---	8,280±50	----	4,445±50	----	2,570±50	----
RB	2,547±20	100	2,075±50	100	1,545±30	100	1,230±30	100
EB	1,415±20	81±5	1,310±30	92±1	830±30	99±1	680±30	99±1
MLB	2,972±20	100	2,150±50	100	1,850±30	100	1,320±20	100

Table R3. The separation performance of GO-TH membranes with different weight ratio of TH to GO.

-	GO-TH (1:1) (60 nm)		GO-TH (1:2) (65±5 nm)		GO-TH (1:3) (65±5 nm)		GO-TH (1:4) (65 ±5 nm)	
	Perm.	Rej.	Perm.	Rej.	Perm.	Rej.	Perm.	Rej.
DW	10,720±30	---	8,530±50	--	6,250±30	---	4,570±30	---
RB	3,612±20	100	2,520±50	100	1,375±30	100	945±30	100
MLB	8,526±30	99±1	6,250±50	100	3,125±30	100	1,250±30	100
EB	10,520±30	67±5	6,730±30	80±3	3,370±30	95±2	2,370±30	98±1

3. Characterization-- Raman is mentioned in the experiment session, but the Raman spectra are not seen in either main text or SI.

Reply: We thank the reviewer very much for kind reminder.

We have removed the related content from experiment section.

-- For the XRD, the reduction peaks in Fig. 1i and Fig. 4c are quite different both in peak position and intensity. As TA, TH are main ingredients of green tea, why their XRD spectra exhibit such huge differences?

Reply: We thank the reviewer very much for kind comment.

The extract of green tea leaves includes 30 – 40% polyphenols (Catechins/TA), 4 – 6% amino acids (*i.e.* TH, glutamic acid, aspartic acid) and the rest (more than 50%) are caffeine, theobromine, theophylline and several other molecules (*J. Funct. Foods*, 2017, 34, 139-151; *Food Res. Int.*, 2009, 42, 529-535). We suggest that the presence of the molecules beyond TA and TH is responsible for the big difference in XRD patterns between GO-TA/TH and GO-GT membranes.

We have added this information in the revised manuscript.

-- Two typos, main text, page 15, line 250, "were s prepared", and SI, caption for SI Fig. 1, "theanine amino acidic"

Reply: We thank the reviewer very much for kind correction. We have checked the manuscript very carefully and corrected all the typos in the revised manuscript.

Reviewer #2 (Remarks to the Author):

Tailoring the structure of graphene oxide (GO) membranes is essentially important for various applications. Recently, GO membranes have been widely demonstrated to have a great potential for water purification, desalination and molecular separation. However, these membranes suffer from a low stability and are easily damaged in aqueous solutions. How to further improve the water permeance without sacrificing the separation efficiency is also very challenging. In this manuscript, the authors fabricated a unique GO-based membranes by using theanine amino acid, tannic acid, and even green tea extractives as cross-linker. Very interestingly, these membranes show alternating GO membrane-like and reduced GO membrane-like domains, which enable excellent stability and superior permeance simultaneously. They show no damage in various aqueous solutions even after months, and water permeation of 10 – 1,000 times higher than those of the reported GO-based membranes and commercial membranes, together with good separation efficiency.

This work is of high novelty and urgent important for not only water treatment but also many solution-phase applications of GO membranes. It also provides valuable information to design rationally 2D nanofluidic channels for selective molecular and ionic transport, which I believe will attract broad interest of 2D material community. Therefore, it can be accepted for publication after minor revision.

Reply: We thank the reviewer very much for positive comments.

1) Please give more detailed information of the GO sheets used for the fabrication of membranes, such as the lateral size, number of layers, etc.

Reply: We thank the reviewer very much for kind suggestion.

The detailed information of the GO sheets has been added in the revised Supplementary Information (Supplementary Fig. 2). The GO sheets are monolayers with a thickness of ~1 nm and lateral size mostly in the range of 0.5 – 1.5 μm.

2) The authors showed that the water permeance of the membranes strongly depends on their thickness. Have the authors checked the effect of the membrane thickness on

the separation efficiency to dyes? Is it possible to further improve the separation efficiency without a great decrease in water permeance by optimizing the thickness of the membranes?

Reply: We thank the reviewer very much for kind suggestion.

According to the reviewer's suggestion, the thickness effect on the separation performance of different dyes (RB, MB and EB) has been investigated using GO-TH membranes with different thickness from ~60 nm to 800 nm. As shown in Table R4, the water permeance decreases while the dye rejection increases with increasing the membrane thickness. The 200-nm-thick membranes show over 10% increase in MB and EB rejection compared to 60-nm-thick membranes and still a very high water permeance that is far better than pristine GO membranes and those reported GO-based membranes. Even for the 800-nm-thick membranes, they still show a water permeance that is better than most of the reported GO membranes but nearly 100% rejection for MB, EB and RB. Therefore, the separation efficiency can be further improved without a great decrease in water permeance by optimizing the thickness of the membranes.

We have added the above discussion and the related data in the revised manuscript.

Table R4. The separation performance of GO-TH membranes with different thickness.

Membrane Thickness (nm)	MB		EB		RB	
	Permeance (LMBH)	Rejection (%)	Permeance (LMBH)	Rejection (%)	Permeance (LMBH)	Rejection (%)
60	10,602±30	71±5	10,520±30	67±5	3,612±20	99±1
100	7,620±30	75±2	7,860±30	71±2	2,045±50	100
200	4,550±30	83±3	5,120±30	80±3	1,520±20	100
400	780 ± 50	95±2	950±30	92± 2	645±20	100
800	275 ± 50	99±1	420±30	98±1	230±20	100

3) The authors are suggested to give more discussions about the origin of the superior water permeance and the different separation efficiency to different dyes of the cross-linked GO-based membranes.

Reply: We thank the reviewer very much for kind suggestion.

The superior water permeance observed in GO-TA and GO-TH membranes is attributed to their unique structure. As reported previously (*Science* 2012, 335, 442-444 and 2014, 343, 752-754), the oxidized regions in GO membranes serve as spacer and graphitic regions allow slip transport. However, the graphitic regions are very limited in pristine GO membranes. Moreover, the pristine GO membranes show a single XRD peak around 11.6° as shown in our manuscript, corresponding to an interlayer distance of ~0.76 nm. As mentioned in our manuscript, reduction and cross-linking take place simultaneously when TA and TH are used. Reduction leads to greatly increased graphitic regions. It is worth noting that both the GO-TA and GO-TH membranes show two XRD peaks. The weak peak is located at around 26.4°, which is attributed to the narrowed interlayer distance of neighboring rGO sheets that are attracted each other by π-π interaction. These rGO membrane domains are not suitable for water permeation because of the very small interlayer distance but play an important role in the stability of membranes. The much stronger XRD peak is located at ~8.9° and 10.4° for GO-TA and GO-TH membranes, respectively, corresponding to interlayer distance of 0.99 nm and 0.85 nm. This indicates that TA or TH molecules are inserted between the neighboring rGO sheets in most of the regions of GO-TA and GO-TH membranes, which act as spacer to ensure a relatively large nanochannel to facilitate water transport. We suggest that the greatly increased graphitic regions and enlarged interlayer distance in these rGO membrane domains are responsible for the much higher water permeance of modified GO membranes than pristine GO membranes. In addition, the covalent bonding between TA/TH molecules and rGO sheets, as shown below, also plays an important role in the stability of the membranes.

As shown in the manuscript, GO-TA and GO-TH membranes show high rejection (~100 %) for cationic dyes such as RB and MLB but low rejection (~67 – 81%) for

anionic dyes such as MB and EB. Note that the GO-TA and GO-TH membranes are negatively charged at neutral pH, therefore, positively charged molecules can be easily taken up by the membranes via electrostatic interactions. As a result, the nanochannels could be partially blocked (*Nat. Commun.* 2013, 4, 2979), leading to a high rejection rate for cationic dyes.

We have added the above discussions in the revised manuscript.

4) There are words misleading in the manuscript. For example, in line 122, there is a word ‘perquisite’, do the authors mean ‘prerequisite’?

Reply: We thank the reviewer very much for kind comment. We have made correction in the revised manuscript.

Reviewers' comments:

Reviewer #1 (Remarks to the Author):

I am very satisfied that the authors have addressed most of my concerns. I have no further questions except the following one,

As the permeance reported in this paper is so high--actually, the highest value I have known. I would be a bit more cautious on this value. Would the authors please, provide a video clip to show the related filtration experiment with record-high permeance together with the reported good rejections?
Linked to this, authors mentioned in the "materials and methods" session, permeance is measured through "a home-made
dead-end filtration device with an effective area of 14.51 cm2 under an applied pressure of 1.0 bar at 296 room temperature. The volume of feed solution was 250 mL. The water permeance was recorded every 10 min until it became steady, and the stable value was recorded as the permeance". I did the caculation, if the permeance is 1034 L/(m2.hr.bar) , 250 ml water could be filtrated out by this setup for 10 min. That is to say, for most of the permeance reported in this manuscript,which is much higher than 1034 L/(m2.hr.bar). For less than 10 min, 250 ml water/dye solution would be filtered out. Has more water/dye solution been added until the permeance became steady in the experiment? if so, abovementioned experiment illustration needs to be adjusted to fit your real experimental details.

Reviewer #2 (Remarks to the Author):

The authors have revised their manuscript based on reviewers' comments. The manuscript can be accepted.

Response to reviewers' comments

Reviewer #1 (Remarks to the Author):

I am very satisfied that the authors have addressed most of my concerns. I have no further questions except the following one.

As the permeance reported in this paper is so high--actually, the highest value I have known. I would be a bit more cautious on this value. Would the authors please, provide a video clip to show the related filtration experiment with record-high permeance together with the reported good rejections?

Reply: We thank the reviewer very much for kind comments.

To show the superior permeability and good rejection of our membranes, we have made videos to record the separation process of RB and MLB through GO-TH membranes (Supplementary Movies 1, 2). 250 mL RB and MLB solutions with a concentration of 50 μM were used as feed, ~60-nm-thick GO-TH membranes were used as filter, the effective filtration area was 11.34 cm^2, and the feed pressure was 1.0 bar. As shown in Supplementary Movies 1 and 2, 250 mL RB and MLB solutions are filtered out by ~230 seconds and ~94 seconds, respectively, which give permeance of ~3450 and ~8440 L m^{-2} h^{-1} bar^{-1}. Moreover, the rejection is ~100% for both cases as confirmed by UV-vis spectra shown in Supplementary Fig. 6. All these results are consistent with those shown in Table 1 in the main text.

These two videos and the corresponding UV-vis spectra have been added in the revised manuscript.

Linked to this, authors mentioned in the "materials and methods" session, permeance is measured through "a home-made dead-end filtration device with an effective area of 14.51 cm2 under an applied pressure of 1.0 bar at 296 room temperature. The volume of feed solution was 250 mL. The water permeance was recorded every 10 min until it became steady, and the stable value was recorded as the permeance". I did the calculation, if the permeance is 1034 L/(m^2.hr.bar) , 250 ml water could be

filtrated out by this setup for 10 min. That is to say, for most of the permeance reported in this manuscript, which is much higher than 1034 L/(m2.hr.bar). For less than 10 min, 250 ml water/dye solution would be filtered out. Has more water/dye solution been added until the permeance became steady in the experiment? if so, abovementioned experiment illustration needs to be adjusted to fit your real experimental details.

Reply: We thank the reviewer very much for kind reminder.

In our experiments, the volume of feed solution was 250 mL for GO, GO-TA and GO-TH membranes and 1,000 mL for GO-GT membranes. For the GO and GO-GT membranes, it usually takes a long time to get a stable water permeance. Therefore, the water permeance was recorded every 10 min for GO and GO-GT membranes until it became steady and the stable value was recorded as the permeance. For the GO-TA and GO-TH membranes, as shown in Supplementary Movies 1 and 2, the permeance is very high and becomes steady very quickly. Therefore, we directly recorded the permeance of GO-TA and GO-TH membranes when the feed pressure reached 1.0 bar until all the solutions were completely filtered out.

We have revised this part and given all the detailed experimental information in the revised manuscript.

REVIEWERS' COMMENTS:

Reviewer #1 (Remarks to the Author):

The videos authors supplemented, very well support the manuscript. I am satisfied with the current version, and recommend for the publication in Nature Communications.

Response to reviewers' comments

Reviewer #1 (Remarks to the Author):

The videos authors supplemented, very well support the manuscript. I am satisfied with the current version, and recommend for the publication in Nature Communications.

Reply: We thank the reviewer very much for the positive comments and kind recommendation.
